# Supplementary material for: Identification of a Quality Marker of Vinegar-Processed Curcuma Zedoaria on Oxidative Liver Injury
Source: Molecules. 2019 May 31;24(11):2073. doi: 10.3390/molecules24112073 (PMC6600333; doi:10.3390/molecules24112073)
Supplement: Supplementary file 1 [file molecules-24-02073-s001.pdf]

Supplementary Information for

Article

Identification of a Quality Marker of Vinegar-Processed Curcuma Zedoaria on Oxidative Liver Injury

Herong Cui [1], Beibei Zhang [1], Guoping Li [1], Lei Li [1], Hongshan Chen [1], Jinchai Qi [1], Wenxue Liu [1], Jing Chen [2,*], Penglong Wang [1,*] and Haimin Lei [1,*]

[1] School of Chinese Pharmacy, Beijing University of Chinese Medicine, Beijing 102488, China; herongcui@outlook.com (H.C.); 20160241011@bucm.edu.cn (B.Z.); zhenzhu7696@163.com (G.L.); wangyq4515@163.com (L.L.); chs1314as@163.com (H.C.); 17862969559@163.com (J.Q.); 18698765005@163.com (W.L.).

[2] Tibetan Traditional Medical College, Lhasa 850000, China.

* Correspondence: cjttmc@sina.cn (J.C.); wpl581@126.com (P.W.); hm_lei@126.com (H.L.)

A list of figures and tables in the Supplementary Information

No.	Headline	Figures/Tables
**1**	Component identification of the crude and processed curcuma zedoaria by GC-MS	Figure S1; Table S1-S4
**2**	The analysis of the fingerprint in the 8 samples of the crude and processed curcuma zedoaria for the multicomponent quality.	Figure S2-S5; Table S5
**3**	The parameters of PCA and OPLS-DA model	Table S6 Figure S6-S7
**4**	The score plots of PCA analysis contained QC samples	Figure S8-S9

1. Component identification of the crude and processed curcuma zedoaria by GC-MS

The chromatographic analysis of component fingerprint was performed on an Agilent 7890B/5977 GCMS system (Agilent Technologies, USA), using a capillary column 5MS (30m × 0.25mm × 0.25 μm). Helium C-60 was used as the carrier gas at a constant flow rate of 1.0 mL/min. Samples (2 μL) were injected in split mode (ratio 1:20) and the injector temperature was 250 °C (held for 20 min). The Gas chromatography settings were as blows. Total separation run time was 93.174 min. The oven temperature was fixed at 60 °C for 1 min, then increasing to 100 °C (rate 4 °C/min), held for 0 min, then increasing to 120 °C (rate 2 °C/min) and held for 0 min, then increasing to 180 °C (rate 1 °C/min), held for 0 min, then increasing to 230 °C (rate 23 °C/min) and held for 10 min. The MS detector was operated in EI mode (70 eV). The EI temperature was 150 °C. Data acquisition was performed in full scan mode with a mass range between 35 and 450 m/z. Sample preparation and GC-MS acquisition were randomized to avoid analytical bias.

The chemical information of the crude and processed curcuma zedoaria was established by GC-MS in the ESI+ modes, and 5, 22, 12, 17 components were tentatively identified by the National Institute of Standards (NIST14) mass spectral libraries (percentages of match of 70 % was set as the accepted mass error), respectively. The mass data and compounds identified from the peaks are summarized in Supplementary **Figure S1 and Table S1-S4**.

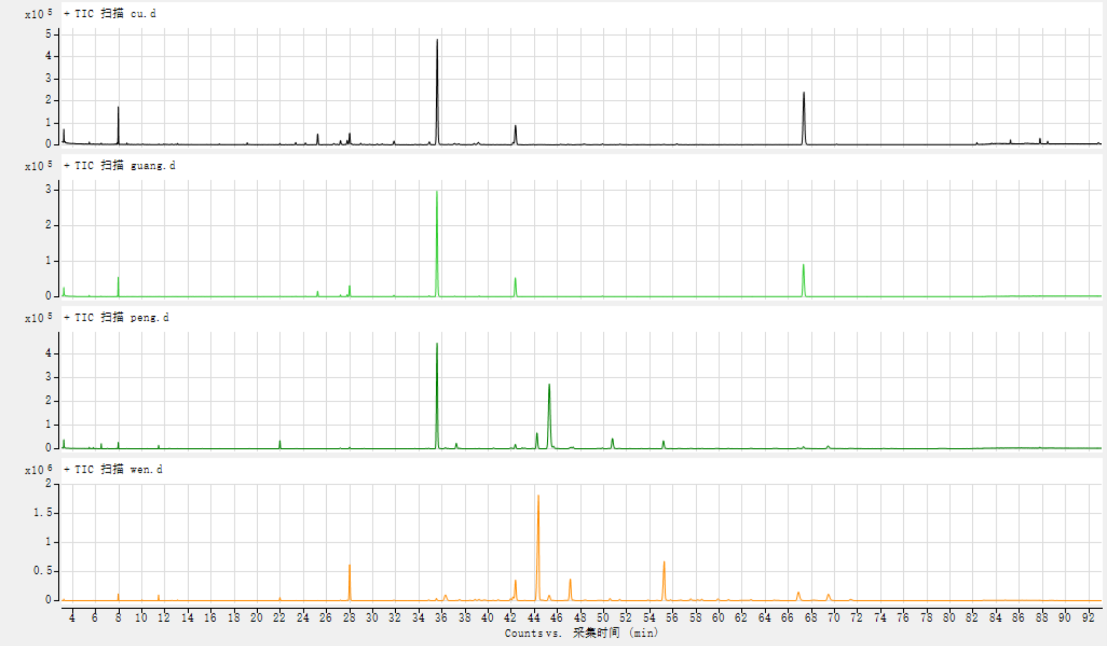

Figure S1 | A base peak chromatogram of the GC-MS analysis of the crude and processed curcuma zedoaria in the ESI+ mode (First: C; Second: G; Third: P; Forth: W). G, W, P, C, the crude (G: *Curcuma kwangsiensis* S. G. Lee et C. F. Liang; W: *Curcuma wenyujin* Y. H. Chen et C.Ling; P: *Curcuma phaeocaulis* Val.) and vinegar-processed (C) curcuma zedoaria.

Table S1 | Identification of components contained in the processed (C) curcuma zedoaria.

No.	RT	Compound Name	Match Factor	Component Area	CAS#	Formula
1	3.24	Trimethylsilylmethanol	81.91	119470	3219-63-4	C4H12OSi
2	5.45	Bicyclo[3.1.0]hex-2-ene, 2-methyl-5-(1-methylethyl)-	81.88	12112	2867-5-2	C10H16
3	7.96	Eucalyptol	98.21	397933	470-82-6	C10H18O
4	8.71	Sulfurous acid, 2-ethylhexyl hexyl ester	86.76	3325	1000309-20-2	C14H30O3S
5	16.71	Oxalic acid, dineopentyl ester	76.58	5845	1000309-72-7	C12H22O4
6	19.13	Cyclohexasiloxane, dodecamethyl-	81.36	22965	540-97-6	C12H36O6Si6
7	25.23	Humulene	82.97	189930	6753-98-6	C15H24
8	27.19	Pyrimidine, 2-trifluoromethylsulfonyl-4,6-dimethyl-	77.20	10338	70371-88-9	C7H7F3N2O2S
9	31.82	Butanoic acid, 3-(1-phenylethoxy)-	73.49	19821	1000195-54-3	C12H16O3
10	35.58	Epicurzerenone	94.96	3152469	20085-85-2	C15H18O2
11	42.36	3,7-Cyclodecadien-1-one, 3,7-dimethyl-10-(1-methylethylidene)-, (E,E)-	85.72	575758	6902-91-6	C15H22O
12	67.34	Zederone	71.46	1420629	7727-79-9	C15H18O3
13	85.23	Cyclononasiloxane, octadecamethyl-	78.84	20814	556-71-8	C18H54O9Si9
14	87.78	Phenol, 2,2'-methylenebis[6-(1,1-dimethylethyl)-4-methyl-	74.20	56638	119-47-1	C23H32O2

Table S2 | Identification of components contained in the crude (G: *Curcuma kwangsiensis* S. G. Lee et C. F. Liang) curcuma zedoaria.

No.	RT	Compound Name	Match Factor	Component Area	CAS#	Formula
1	3.24	Ethane, 1,1-diethoxy-	77.82	57388	105-57-7	C6H14O2
2	7.95	Eucalyptol	91.96	114425	470-82-6	C10H18O
3	35.55	Epicurzerenone	92.06	1867777	20085-85-2	C15H18O2
4	42.35	3,7-Cyclodecadien-1-one, 3,7-dimethyl-10-(1-methylethylidene)-, (E,E)-	75.90	277578	6902-91-6	C15H22O
5	67.29	Zederone	80.26	419917	7727-79-9	C15H18O3

Table S3 | Identification of components contained in the crude (W: *Curcuma wenyujin* Y. H. Chen et C.Ling) curcuma zedoaria.

No.	RT	Compound Name	Match Factor	Component Area	CAS#	Formula
1	3.23	Ethane, 1,1-diethoxy-	79.11	62358	105-57-7	C6H14O2
2	5.79	2-Oxo-3-pyrazin-2-ylpropionic acid, ethyl ester	70.57	3272	1000188-00-0	C9H10N2O3
3	6.98	5-Hydroxy-7-methoxy-2-methyl-3-phenyl-4-chromenone	81.30	1249	55927-39-4	C17H14O4
4	7.95	Eucalyptol	92.77	252071	470-82-6	C10H18O
5	11.44	(+)-2-Bornanone	95.54	254939	464-49-3	C10H16O
6	13.09	.alpha.-Terpineol	73.87	25968	98-55-5	C10H18O
7	21.96	Cyclohexane, 1-ethenyl-1-methyl-2,4-bis(1-methylethenyl)-	84.38	173255	110823-68-2	C15H24
8	28.00	Benzofuran, 6-ethenyl-4,5,6,7-tetrahydro-3,6-dimethyl-5-isopropenyl-, trans-	97.69	3422848	17910-09-7	C15H20O
9	35.50	beta.-Elemenone	78.03	164256	20303-60-0	C15H22O
10	36.29	gamma.-Elemene	73.26	960137	29873-99-2	C15H24
11	39.22	Imidazo[4,5-d]imidazole, 1,6-dihydro-	77.59	35848	35369-36-9	C4H4N4
12	42.36	3,7-Cyclodecadien-1-one, 3,7-dimethyl-10-(1-methylethylidene)-, (E,E)-	95.16	2694066	6902-91-6	C15H22O
13	44.35	Curdione	98.59	17191574	13657-68-6	C15H24O2
14	45.28	Curcumenol	75.66	675174	19431-84-6	C15H22O2
15	47.11	Curdione	80.70	2122126	13657-68-6	C15H24O2
16	55.24	Curcumenone	97.71	6032333	100347-96-4	C15H22O2
17	58.47	Bicyclo[3.2.2]nona-6,8-dien-3-one	71.86	43127	26788-91-0	C9H10O
18	60.83	Benzenemethanol, 4-methyl-.alpha.-(1-methyl-2-propenyl)-, (R*,R*)-	73.48	59395	83173-76-6	C12H16O
19	71.41	Propanedioic acid, dihydroxy-, bis(1-methylethyl) ester	77.76	70030	70841-85-9	C9H16O6

Table S4 | Identification of components contained in the crude (P: *Curcuma phaeocaulis* Val.) curcuma zedoaria.

No.	RT	Compound Name	Match Factor	Component Area	CAS#	Formula
1	3.23	1,2-Propanediol, 3-methoxy-	80.88	59047	623-39-2	C4H10O3
2	6.48	Bicyclo[3.1.0]hexane, 4-methylene-1-(1-methylethyl)-	77.62	31353	3387-41-5	C10H16
3	6.98	5-Hydroxy-7-methoxy-2-methyl-3-phenyl-4-chromenone	81.35	2287	55927-39-4	C17H14O4

4	7.95	Eucalyptol	84.44	48029	470-82-6	C10H18O
5	11.45	(+)-2-Bornanone	81.11	26418	464-49-3	C10H16O
6	21.96	Cyclohexane, 1-ethenyl-1-methyl-2,4-bis(1-methylethenyl)-	79.05	104686	110823-68-2	C15H24
7	35.56	Epicurzerenone	93.58	2881930	20085-85-2	C15H18O2
8	42.35	4,6-Bis(4-ethoxybenzylthio)-5-nitropyrimidine	70.76	3198	325957-76-4	C22H23N3O4S2
9	44.21	Curdione	81.66	382821	13657-68-6	C15H24O2
10	45.28	Curcumenol	76.42	2178843	19431-84-6	C15H22O2

2. **The analysis of the fingerprint in the 8 samples of the crude and processed curcuma zedoaria for the multicomponent quality.**

Eight samples of curcuma zedoaria (four varieties) was purchased from China pharmaceutical biological products inspection institute, China. The chromatographic analysis of component fingerprint was performed as above mentioned. The professional software "Similarity Evaluation System for Chromatographic Fingerprint of Traditional Chinese Medicine" (Version 2004A, SES software) and Origin Pro (Version 8.5, OriginLab software) was used for evaluating the similarities between samples. The reference chromatogram was generated with average data. Sample no. 1 was selected as a representative sample to validate the method for fingerprint analysis. A fingerprint of curcuma zedoaria was then established (Supplementary **Figure S2**). Supplementary **Table S5** shows the similarity values of each sample. The closer the similarity value was to 1, the more similar was the chromatogram to the reference chromatogram. As is shown in **Table S5**, the similarity values of all 8 samples in respective variety of curcuma zedoaria were higher than 0.985, using the angle cosine method based on common peak by the similarity evaluation system (A version of traditional Chinese medicine chromatographic fingerprint).

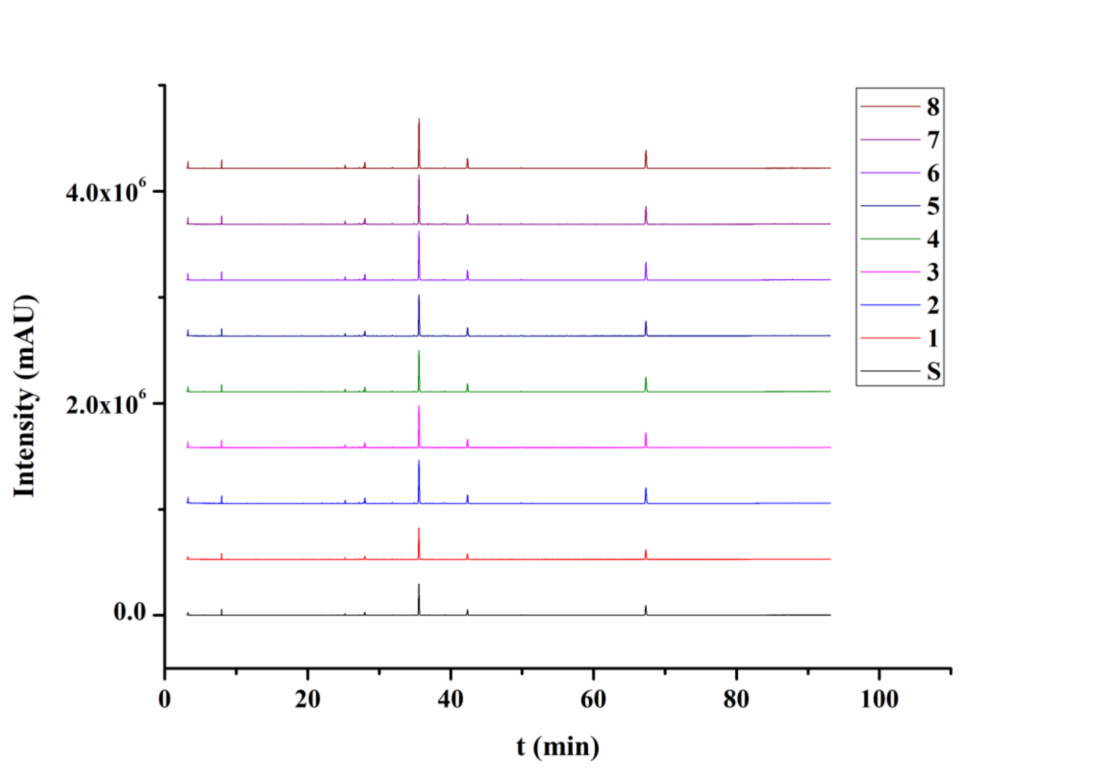

Figure S2 | Chemical fingerprint of the crude (G: *Curcuma kwangsiensis* S. G. Lee et C. F. Liang) curcuma zedoaria nos. 1-8 (S1-S8) were used to construct a fingerprint.

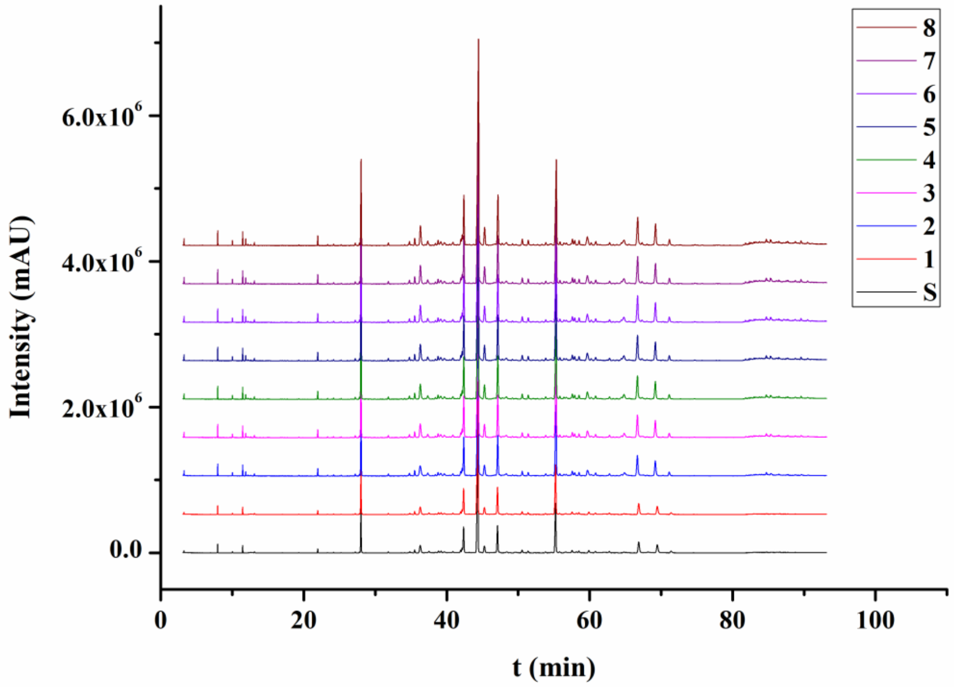

Figure S3 | Chemical fingerprint of the crude (W: *Curcuma wenyujin* Y. H. Chen et C.Ling) curcuma zedoaria nos. 1-8 (S1-S8) were used to construct a fingerprint.

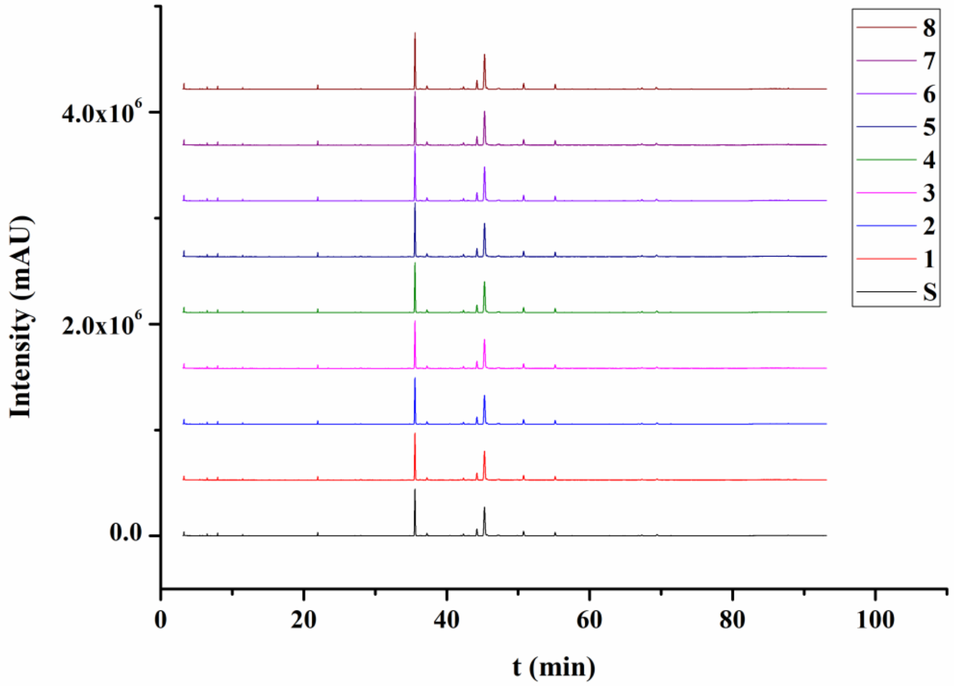

Figure S4 | Chemical fingerprint of the crude (P: *Curcuma phaeocaulis* Val.) curcuma zedoaria nos. 1-8 (S1-S8) were used to construct a fingerprint.

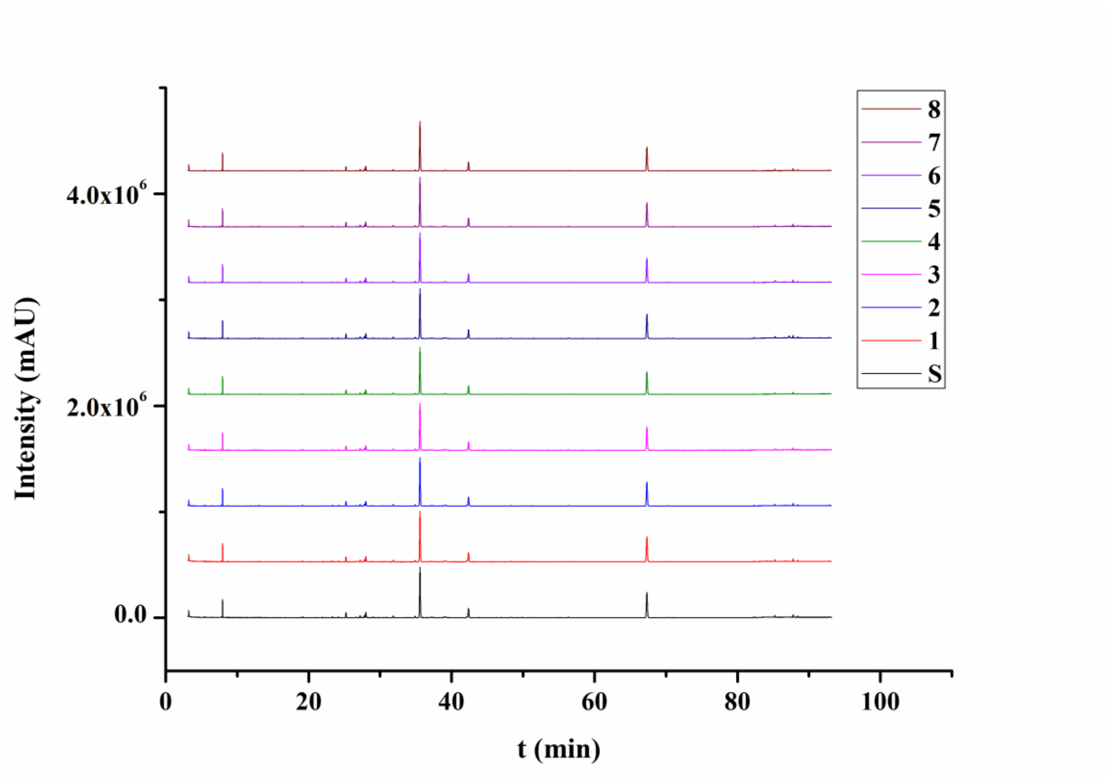

Figure S5 | Chemical fingerprint of the processed (C) curcuma zedoaria nos. 1-8 (S1-S8) were used to construct a fingerprint.

Table S5 | The similarities of chromatograms of each sample

G		W		P		C	
No.	Similarity	No.	Similarity	No.	Similarity	No.	Similarity
1	1.000	1	1.000	1	1.000	1	1.000
2	0.988	2	0.992	2	0.989	2	0.993
3	0.987	3	0.991	3	0.988	3	0.992
4	0.988	4	0.992	4	0.991	4	0.991
5	0.991	5	0.990	5	0.991	5	0.989
6	0.992	6	0.989	6	0.993	6	0.991
7	0.990	7	0.991	7	0.989	7	0.988
8	0.985	8	0.990	8	0.990	8	0.994

G, W, P, C, the crude (G: *Curcuma kwangsiensis* S. G. Lee et C. F. Liang; W: *Curcuma wenyujin* Y. H. Chen et C.Ling; P: *Curcuma phaeocaulis* Val.) and vinegar-processed (C) curcuma zedoaria.

3. The parameters of PCA and OPLS-DA model

Table S6 | The parameters of PCA and OPLS-DA model

Model	Component	R2X	R2Y	Q2Y	R2-intercept	Q2-intercept
PCA (M1)	2	0.303	—	-0.033	—	—
PLS-DA (M2)	3+1+0	0.469	0.965	0.879	0.081	0.250

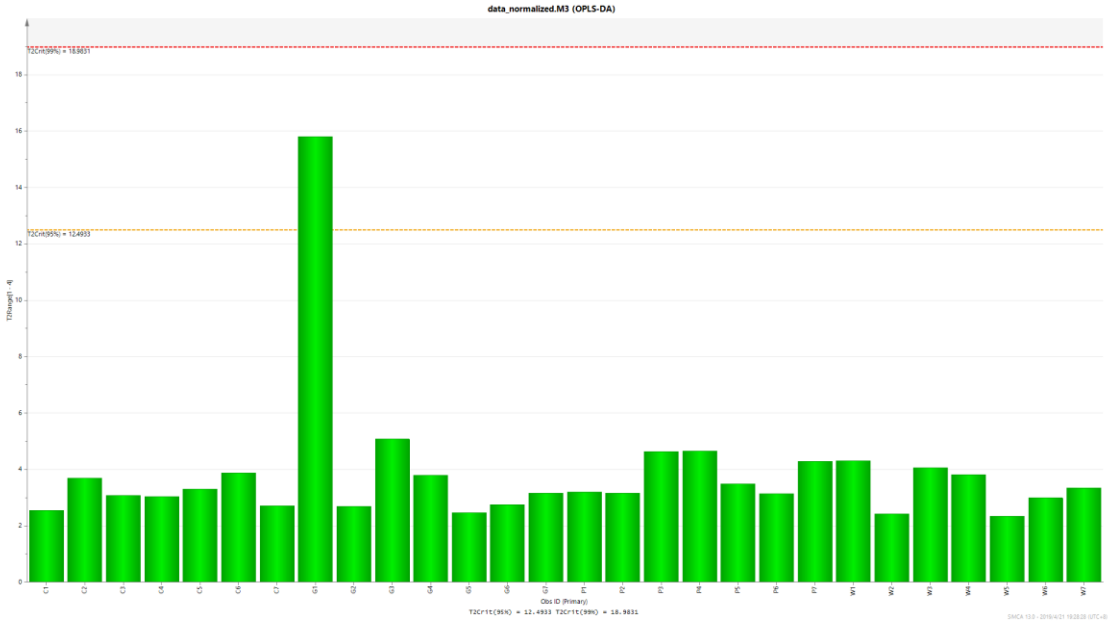

Figure S6 | Hotelling's T2 of OPLS-DA (M2).

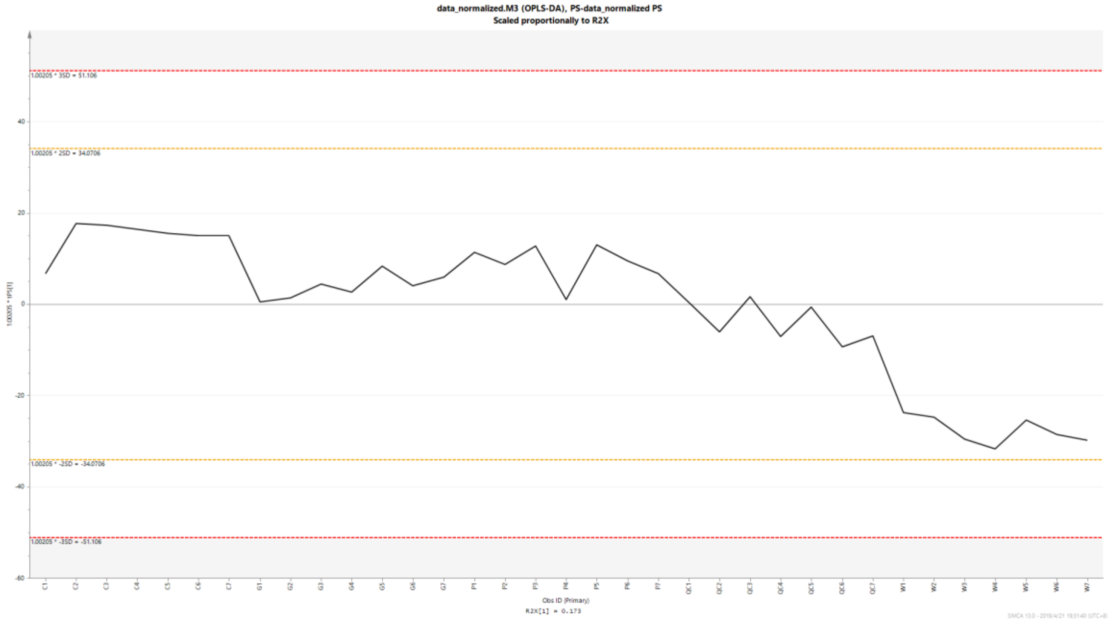

Figure S7 | Score plot (B) of OPLS-DA (M2).

4. The score plots of PCA analysis contained QC samples

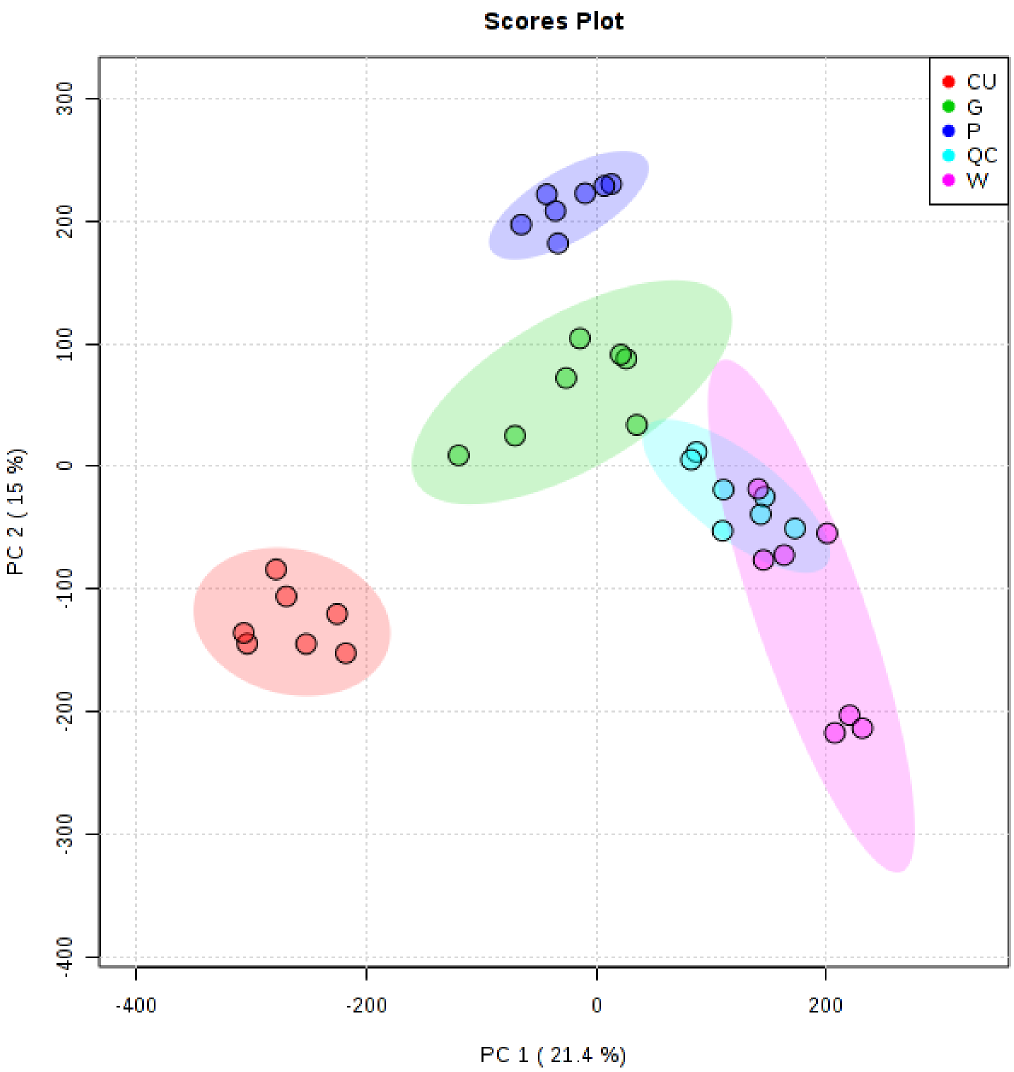

Figure S8 | The score plots of PCA analysis contained QC samples. G, W, P, CU, the crude (G: *Curcuma kwangsiensis* S. G. Lee et C. F. Liang; W: *Curcuma wenyujin* Y. H. Chen et C.Ling; P: *Curcuma phaeocaulis* Val.) and vinegar-processed (CU) curcuma zedoaria.

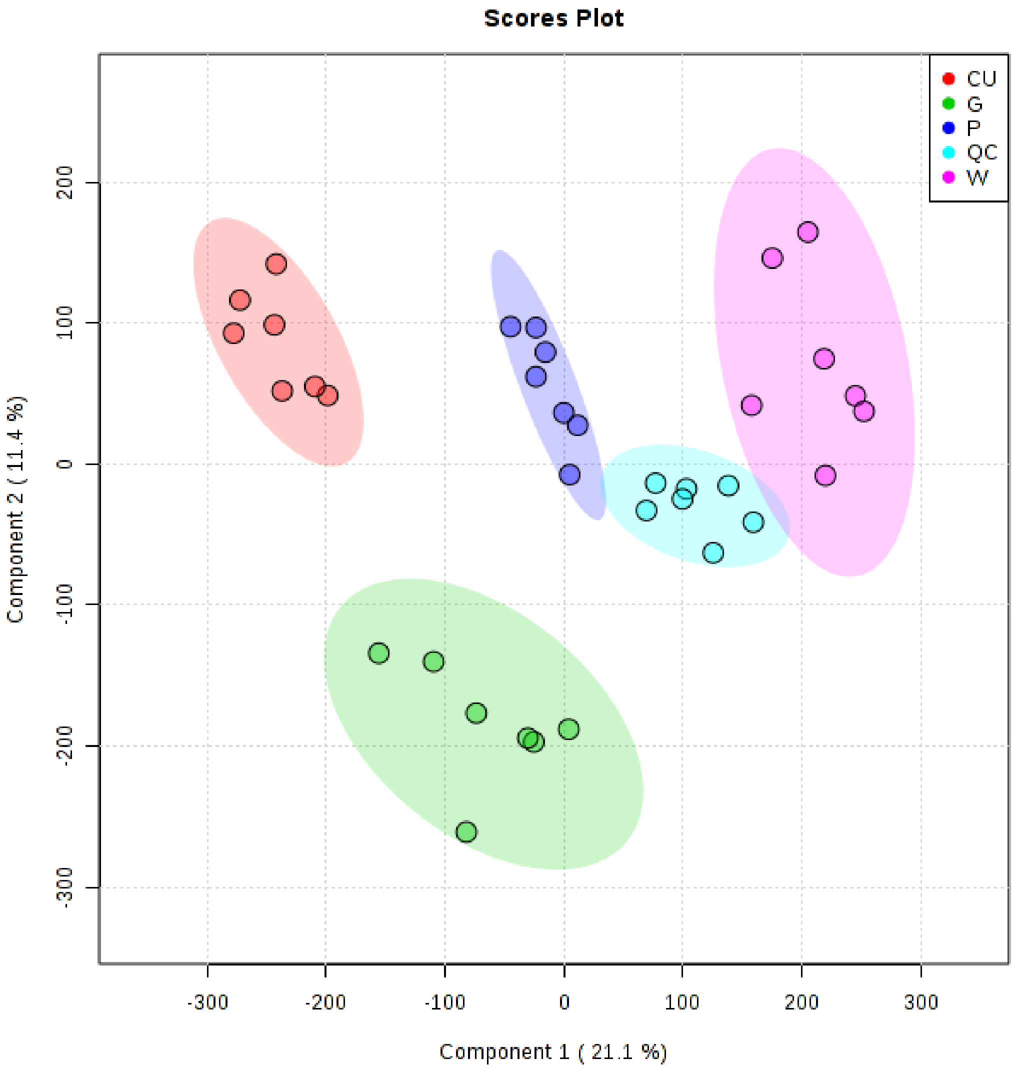

Figure S9 | The score plots of OPLS-DA analysis contained QC samples. G, W, P, CU, the crude (G: *Curcuma kwangsiensis* S. G. Lee et C. F. Liang; W: *Curcuma wenyujin* Y. H. Chen et C.Ling; P: *Curcuma phaeocaulis* Val.) and vinegar-processed (CU) curcuma zedoaria.
